# Objective performance indicators during robotic Roux-en-Y gastric bypass distinguish experienced from trainee surgeons: a cross-sectional study

**DOI:** 10.1007/s00464-025-11873-4

**Published:** 2025-07-14

**Authors:** Wendy S. Li, Qais AbuHasan, Dimitrios I. Athanasiadis, Andrew Yee, Dimitrios Stefanidis

**Affiliations:** 1 Department of Surgery, Indiana University School of Medicine, Indianapolis, IN USA; 2 Data and Analytics, Intuitive Surgical, Inc, Peachtree Corners, GA USA; 3 Department of Surgery, Indiana University School of Medicine, 545 Barnhill Drive, EH 122, Indianapolis, IN 46202 USA

**Keywords:** Objective performance indicators, Robotic surgery, Kinematics, Resident training, Bariatric surgery, Roux-en-Y gastric bypass

## Abstract

**Introduction:**

Objective performance indicators (OPIs) obtained during robotic surgery have been associated with surgeons’ experience and may influence patient outcomes. However, their evaluation in bariatric surgery has received little attention. The study aimed to evaluate whether OPIs can effectively distinguish experienced surgeons from trainees during robotic Roux-en-Y gastric bypass (r-RYGB).

**Methods:**

Kinematic and event data were recorded during r-RYGB operations (*n* = 31; 2 experienced attending surgeons, 6 trainees) utilizing the da Vinci Surgical System’s data recorder between August 2021 and December 2022. De-identified data were extracted, videos annotated within eight surgical steps, and OPI generated and analyzed. OPIs generated included console active time, instrument movement speed (path length/console active time), bimanual dexterity (ratio of dominant to non-dominant instrument path length), and workspace volume rate.

**Results:**

Significant OPI differences were noted in 3 out of 8 steps of the procedure. During the dissection phase, attending surgeons exhibited greater right-hand controller usage (bimanual dexterity, *p* = 0.018), and faster non-dominant instrument path length speed (*p* = 0.002). During the creation of the gastric pouch, experts displayed similar higher bimanual dexterity (*p* = 0.001), but slower non-dominant instrument path length speed (*p* = 0.012). Trainees had a lower active console time than attending surgeons during gastric pouch creation (*p* = 0.03) and hand-sewn anastomosis (*p* = 0.005). Both cohorts demonstrated similar OPIs during other steps such as mesenteric division, mesenteric closure, common channel enterotomy closure, limb measurement, and stapled anastomosis.

**Conclusion:**

Step-specific OPIs obtained during robotic surgery can effectively distinguish between experienced and trainee surgeons. With further validation, OPIs may provide an objective assessment of trainee performance and aid in training and autonomy decisions.

**Supplementary Information:**

The online version contains supplementary material available at 10.1007/s00464-025-11873-4.

Surgical skill has been shown to directly impact patient outcomes [1–4]. Accurate assessment of surgical skill is paramount as it can enable operative performance improvement using learnings from objective feedback. In robotic surgery, survey assessment tools, such as GEARS and R-OSATS, have been developed to measure surgeon skill and determine competency [5–7]. Unfortunately, such tools are subject to multiple rater biases, significant rating variability, and low completion rates that erode their value [8, 9]. The computerized platform of robotic surgery allows for the extraction of objective and quantitative metrics that can be utilized to assess operative performance, which are known as objective performance indicators, OPIs [10–12].

Previous studies have shown that OPIs can be associated with surgeons’ experience in multiple surgical specialties. In urology, measurable differences in OPIs between surgeons of varying experience levels were detected during different steps of robot-assisted radical prostatectomy, RARP [13]. Similarly, during thoracic lobectomy, studies have found differences in OPIs in both exposure and stapling tasks between more and less experienced surgeons [14, 15]. Furthermore, in general surgery, OPIs for camera control, and master clutch and arm swap were found to be distinctively different between fellows and attending surgeons during cholecystectomy [16]. Robotic surgery utilization in bariatrics has been steadily increasing since 2015, with about one-third of bariatric procedures being performed robotically [17]. While OPIs have been investigated in other surgical fields, their utility in bariatric surgery has yet to be explored. OPIs could be used to assess specific surgeon performance and provide feedback for learners in bariatric surgery.

In this study, we aimed to assess the ability of OPIs to effectively distinguish between experienced surgeons and trainees during key steps of a robotic Roux-en-Y gastric bypass (r-RYGB).

## Methods

### Data collection

Following approval from the Institutional Review Board (IRB#2012912159) as an expedited study, we conducted a cross-sectional study using kinematic and event data recorded by the Intuitive data recorder during r-RYGB operations utilizing the da Vinci Surgical System Xi between August 2021 and December 2022. The cases took place at a bariatric center of excellence in a large academic institution. Participants included attending bariatric surgeons, minimally invasive surgery (MIS) fellows, and general surgery residents. Informed consent to record cases was obtained from all participants. Patient data, including patient demographics (age, race, gender), preoperative comorbidities, body mass index, and American Society of Anesthesiologists (ASA) class were retrospectively extracted from electronic medical records. Perioperative data, including operative duration and post-operative information such as length of stay, were also documented. Cases in which trainees did not participate at the robotic console were excluded from the study. Surgeon and trainee demographic and experience information were recorded.

### Kinematic-based metrics and OPI calculations

De-identified data generated from the da Vinci surgical system and recorded via the Intuitive data recorder were sent to Intuitive for data extraction and generation of OPIs, which were subsequently returned to our institution for further analysis. Time-related metrics were captured in seconds, which included the total duration of steps, the movement time of both the dominant and non-dominant console hand controllers, and the percentage of active console time. Console-based metrics included instrument pathlength speed of both hands (cm/s), bimanual dexterity (ratio of dominant to non-dominant instrument pathlength), and workspace volume (cm^3^). To eliminate the influence of duration on workspace volume and ensure unbiased comparison, we normalized workspace volume with respect to time, resulting in a time-adjusted workspace volume metric (cm^3^/s). We then cross-verified this normalization by also adjusting for the path length traveled, confirming that our evaluation of workspace efficiency is consistent across both time and instrument movement parameters. The descriptions and calculations of the OPIs can be found in Table 1. The focus on time and console motion-related metrics were based on previous studies that have reviewed and validated these metrics as effective indicators for performance [18, 19].

**Table 1:** Overview of OPI and description

Objective performance indicator	Description
Time-related metrics	
Total task duration (s)	Total time taken to complete a specific portion of procedure recorded in seconds
Active console time (s)	Active time surgeon is actively manipulating instrument using the console
Percentage of active console time (%)	Proportion of the total task duration during which each console was active
Console-based metrics	
Instrument pathlength (cm)	Total distance traveled by the instrument based on the console
Instrument pathlength speed (cm/s)	Average speed at which the instruments are moving during active console time
Bimanual dexterity	Ratio of the dominant hand instrument pathlength to the non-dominant hand instrument pathlength
Workspace volume (cm^3^)	Total volume of space occupied by the movement of the surgical instruments in three dimensions^a^
Workspace volume per time (cm^3^/s)	Average volume space occupied by instrument movement per second of active console time

^a^Console sensitivity was tested and was consistent among surgeons by verifying the ratio of instrument path length and the console wrist path length

### Ontology of surgical steps

**Fig. 1:**
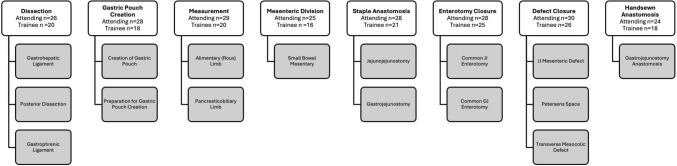
Division of operative steps for the r-RYGB cases. The *n* represents the number of cases included for analysis for attending and trainees for each surgical step

The specific surgical steps during r-RYGB were organized and grouped into eight key steps to facilitate a more precise comparison of OPIs (Fig. 1). The organization involved combining subtasks, as well as smaller, segmented tasks into these eight main surgical steps for similar comparison. The steps were also organized based on the sequence they occurred during the procedure, ensuring that the steps aligned with the natural progression of the surgery. The grouping enabled a focused analysis on steps that demand similar technical skills and maneuvers. Steps with inherently smaller sample sizes, such as the leak test and concurrent hiatal hernia repair, and those without both console data were excluded from the analysis to ensure the reliability and relevance of the data, thus ensuring that the OPIs evaluated are focused on the most critical aspects of the surgical procedure.

### Statistical analysis

Descriptive analysis was reported using frequency for categorical variables and median and means for continuous variables depending on the normality of the data. Continuous values were reported as marginal mean (95% confidence interval) for normally distributed data and median with interquartile range (IQR) for nonparametric data. For the assessment of OPIs, the attending surgeon and trainee console metrics were compared using independent *t* tests between the groups for normally distributed variables. We also compared OPIs between less and more experienced trainees (i.e., residents and fellows, respectively). Mann–Whitney test was used for nonparametric data. *p* < 0.05 was considered significant. All analyses were done using IBM SPSS Statistics vs. 29.0 (IBM Corp., Armonk, NY, USA).

## Results

### Patient and surgeon characteristics

Forty-nine r-RYGB procedures were performed and recorded between August 2021 and December 2022. Patient characteristics and intraoperative data (video, kinematics, and events) were available for 31 cases. The average age of the patient was 47.4 ± 11 years. The majority of procedures (74.2%) were primary bariatric operations followed by revisions. Complete patient demographic data are listed in Table 2. The console kinematic and event data included information from 2 experienced surgeons with 500 and 1300 prior robotic operations and 6 trainee surgeons with median cases of 17.5 (range 3–125). Trainees consisted of 4 residents and 2 MIS fellows. All surgeons self-reported as right-hand dominant. There were no statistically significant differences in patient characteristics and case complexity between resident and fellow-involved cases.

**Table 2:** Patient demographics, procedural details, and surgeon characteristics of included r-RYGB cases

Variable	*n* (%) or mean ± SD
Age (years)	47.4 ± 11
Race	
White	24 (77.4%)
Black or African American	6 (19.4%)
Unknown/not reported	1 (3.2%)
Gender	
Female	26 (83.9%)
Male	5 (16.1%)
BMI (kg/m^2^)	47.9 ± 7
Smoking status	
Non-smoker	31 (100%)
Smoker	0
Diabetes status	
Not diabetic	21 (67.7%)
Diabetic	10 (32.3%)
Hypertension	
No	11 (35.5%)
Yes	20 (64.5%)
ASA classification	
Class II	2 (6.5%)
Class III	27 (87.1%)
Class IV	2 (6.5%)
Length of stay (days)	1.9 [1, 2]^a^
Type of procedure	
Initial	23 (74.2%)
Conversion	8 (25.8%)
Operation time (h)	3.5 ± 0.8
Trainee	6
Robotic case experience	17.5 (3, 125)
Attending surgeon	2
Robotic case experience	600 (500, 1300)
Right hand dominance	8

^a^Represent nonparametric data reported as median [IQR]

### OPI differences among experienced and trainee surgeons

Console active time during various steps of r-RYGB revealed that attending surgeons generally had a higher percentage of active console time when compared to trainees (Table 3). During gastric pouch creation, attending surgeons had a significantly higher total active console time (78.7% vs. 56.1%, respectively; *p* = 0.03) and dominant hand active time (61.3% vs. 27.3%, respectively; *p* < 0.001) when compared with trainees. Similarly, during hand-sewn anastomosis, attending surgeons had significantly more active time (72.9% vs. 40.8%, respectively; *p* = 0.005) and dominant hand usage (66.2% vs. 37.1%, *p* = 0.004). However, although not statistically significant, trainees exhibited a slightly higher percentage of active console time (69.6% vs. 65.1%, *p* = 0.674) during defect closure.

**Table 3:** Median active console time comparing attending surgeon with trainee

	Total average task duration (s)	Total active console time (%)			Non-dominant hand active time (%)			Dominant hand active time (%)		
		Trainee	Attending	*p*	Trainee	Attending	*p*	Trainee	Attending	*p*
Dissection	311.7 ± 178.1	41.3 [21.5, 88.8]	89.7 [58.5, 92.6]	0.086	36.7 [18.6, 73.7]	64.6 [40.3, 75.5]	0.212	22 [6.12, 62.1]	67.4 [39.6, 80.7]	**0.023**
Creation of gastric pouch	690.7 ± 307.5	56.1 [37.9, 77.1]	78.7 [69.1, 85.3]	**0.03**	49.1 [23.6, 66.9]	61 [50.8, 65]	0.234	27.3 [18.8, 51.7]	61.3 [37.4, 66.8]	** < .001**
Measurement	212.3 ± 103.2	80.1 [60.5, 93.7]	91.5 [51.2, 97.4]	0.354	73.3 [56, 83.1]	79.4 [44.9, 86.4]	0.412	66.5 [10.9, 85.5]	77.2 [25.1, 88.9]	0.315
Division of mesentery	97.8 ± 78.2	77 [71.1, 81.2]	84.8 [74.1, 88.6]	**0.018**	67.8 [50.8, 79.6]	63.1 [51.9, 76.8]	0.76	21.3 [15, 62.8]	64 [44.8, 82.4]	**0.004**
Staple anastomosis	336.9 ± 85.4	71 [65.1, 73.4]	78.8 [66.9, 83.4]	0.074	59.3 [68.2, 76]	63.6 [47.2, 72.3]	0.4	43.3 [19.9, 59.5]	57.1 [71.8, 77.6]	0.06
Enterotomy closure	742.3 ± 300.5	64.7 [41.7, 92.9]	68.8 [57.1, 92.1]	0.495	50.6 [31.8, 72]	53.5 [33.4, 69.7]	0.86	53 [33.5, 82.4]	61.7 [47.1, 72.3]	0.455
Defect closure	880.8 ± 311.7	69.6 [57.5, 78.7]	65.1 [48.5, 88.6]	0.674	52.3 [40.8, 69.2]	44.6 [29.7, 69.6]	0.411	61.4 [50.7, 70.9]	55.6 [42.1, 77]	0.674
Hand-sewn anastomosis	1959 ± 28.1	40.8 [30, 69]	72.9 [51.2, 97.4]	**0.005**	32.4 [25.2, 44.4]	49.8 [45.3, 65.2]	**0.002**	37.1 [27.5, 62]	66.2 [ 47.2, 79]	**0.004**

Bold value indicates statistical significance

Average duration is reported as marginal mean +/− standard deviation. Percentage values are represented as median [IQR] and *p* values are calculated via Mann–Whitney *U* test

**Fig. 2:**
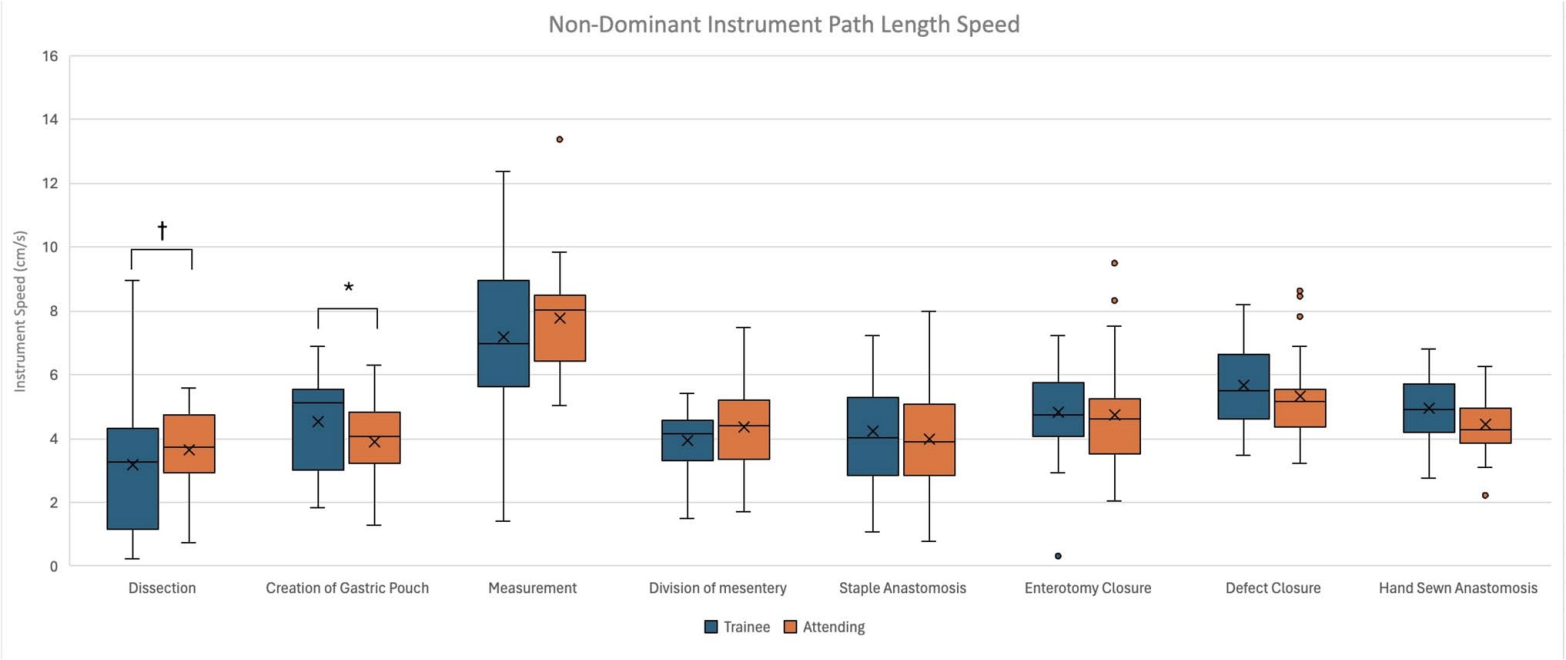
Non-dominant instrument linear speed categorized by operative steps comparing trainee and attending surgeons, line within graph indicates cohort median, while *x* represents the mean. ^†^Indicates statistical significance with *p* value < − 0.05 using the Mann–Whitney *U* test for nonparametric data, while *indicates significance for normally distributed data, and the two groups were compared using independent *t* test

**Fig. 3:**
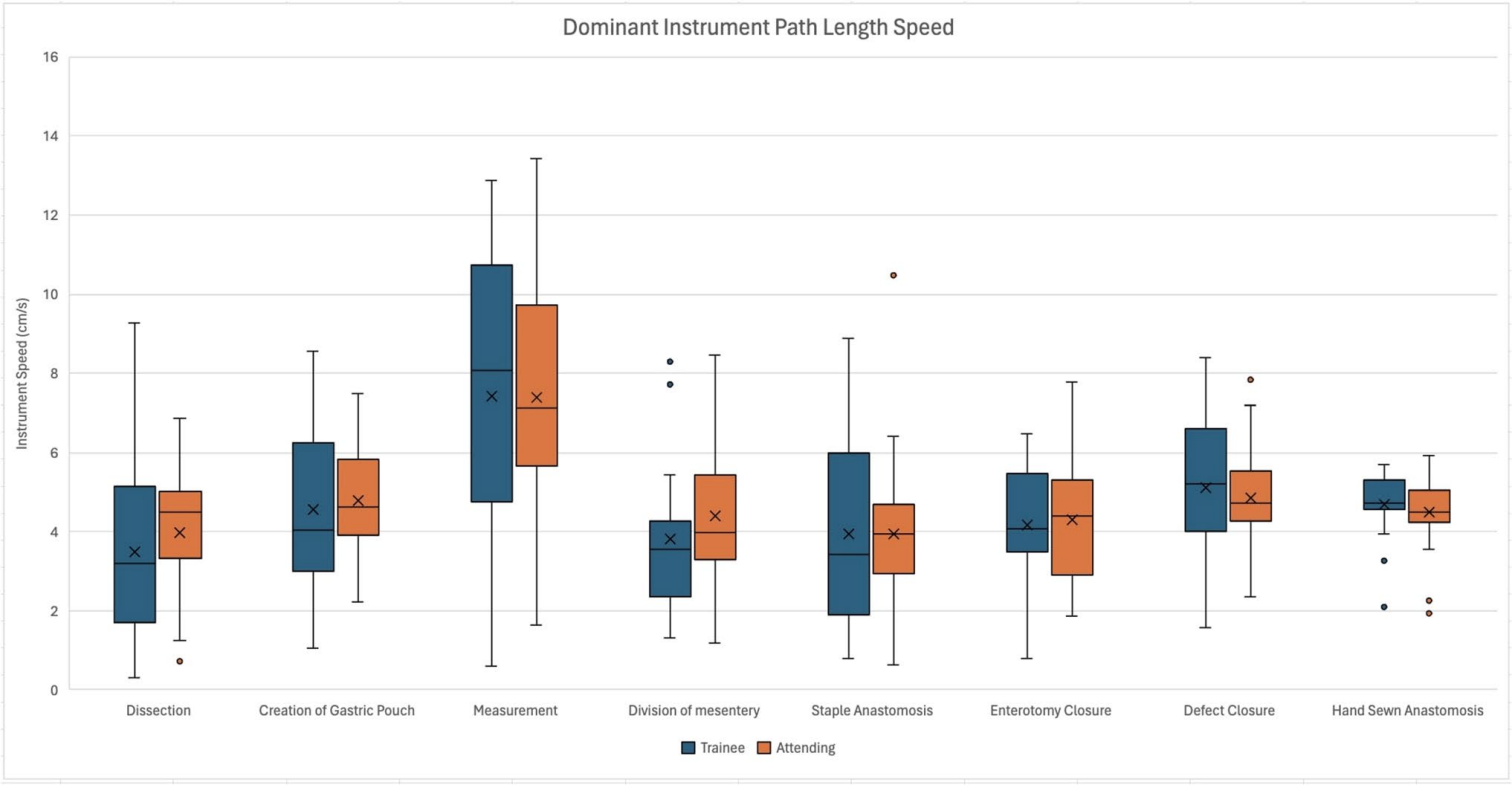
Dominant instrument linear speed categorized by operative steps comparing trainee and attending surgeons, line within graph indicates cohort median, while *x* represents the mean

**Fig. 4:**
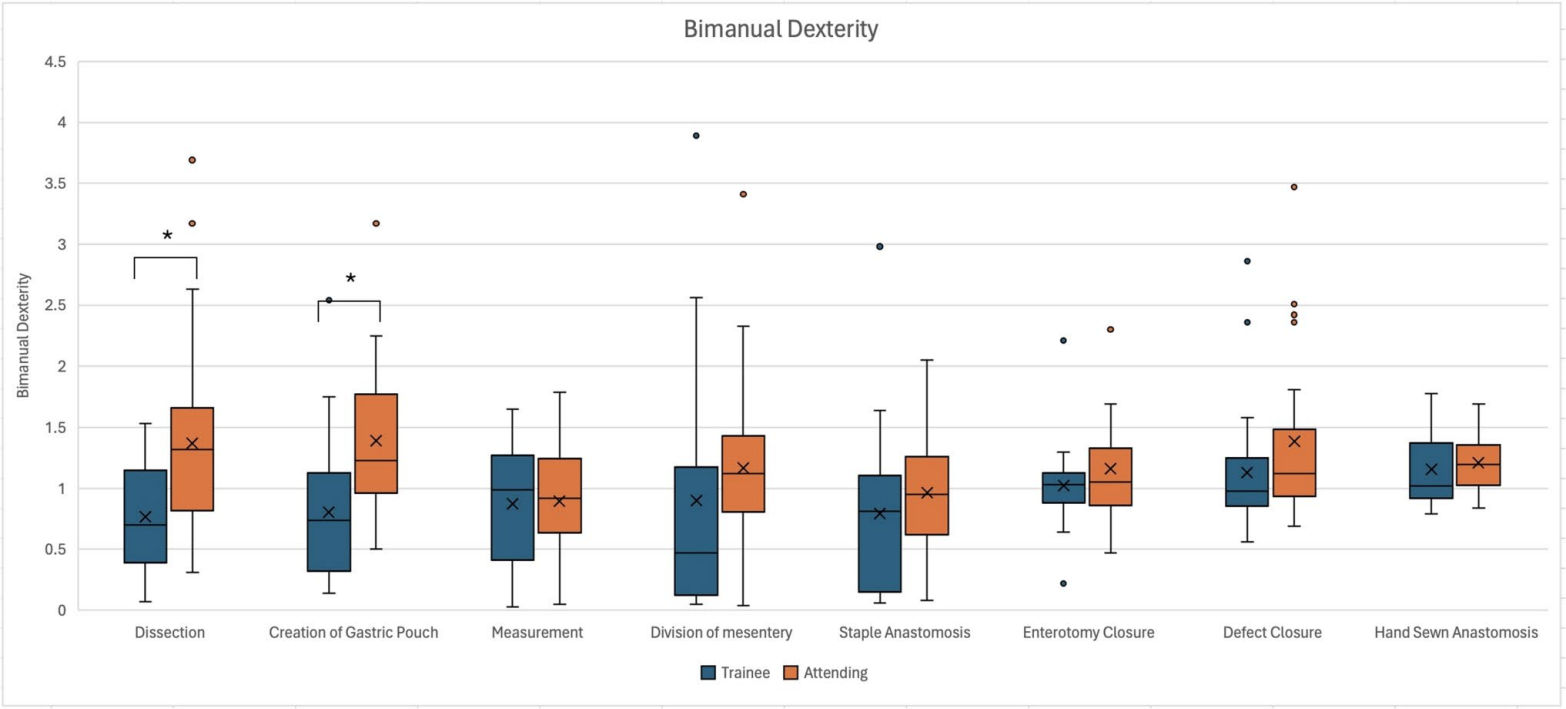
Bimanual dexterity OPI categorized by operative steps comparing trainee and attending surgeons, line within graph indicates cohort median, while *x* represents the mean. *Indicates statistical significance between two groups with *p* value < 0.05 for normally distributed data

**Fig. 5:**
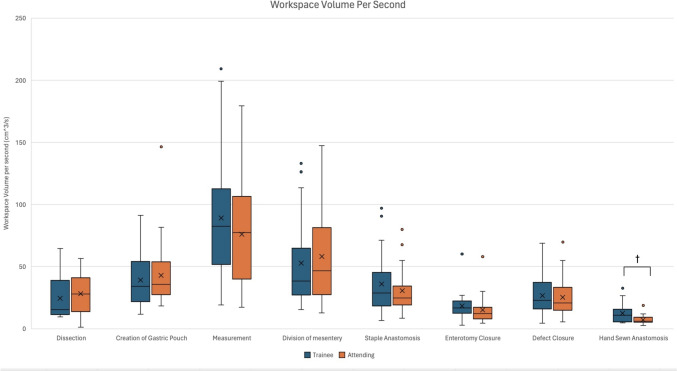
Normalized workspace volume with respect to time categorized by operative steps comparing trainee and attending surgeons, line within graph indicates cohort median, while *x* represents the mean. ^†^Indicates statistical significance with *p* value < − 0.05 using the Mann–Whitney *U* test for nonparametric distributed data

In the analysis of additional console-based metrics during various steps of r-RYGB, some distinct differences were seen. The non-dominant instrument path length speed was faster for the attending surgeons when compared to trainees (3.74 cm/s vs. 3.25 cm/s, *p* = 0.002) during the dissection steps of the case, while during gastric pouch creation, the non-dominant instrument linear speed was slower for the attending surgeons compared to trainees (3.91 cm/s vs. 4.52 cm/s, *p* = 0.012, Fig. 2). Although not statistically significant, the attending surgeon cohort had faster dominant instrument speed during the steps of dissection, creation of gastric pouch, division of mesentery, and staple anastomosis (Fig. 3). With regard to bimanual dexterity, attending surgeons had higher bimanual dexterity across all steps and were statically significant during dissection (1.37 vs. 0.71, *p* = 0.018) and creation of the gastric pouch (1.23 vs. 0.73, *p* = 0.001) when compared to trainees (Fig. 4). In the hand-sewn anastomosis step, attending surgeons showed a significant decrease in normalized total workspace volume (5.96 cm^3^/s vs. 10.93 cm^3^/s, *p* = 0.027, Fig. 5). When the workspace volume was normalized using the path length of the instrument, a similar statistical finding was observed, reinforcing the consistency of the metric (Supplemental Table 1).

### Training level subgroup analysis

Looking at resident performance specifically, the instrument path length speed was slower when compared to attending surgeons for both dominant and non-dominant hands across the steps of the r-RYGB (Table 4). Attendings had faster non-dominant instrument path length speed during measurement (7.77 cm/s vs. 4.49 cm/s, *p* < 0.001) and dissection (3.66 cm/s vs. 2.33 cm/s, *p* = 0.023) and faster dominant instrument speed during mesenteric division (4.41 cm/s vs. 2.49 cm/s, *p* = 0.02) and dissection (3.99 cm/s vs. 1.91 cm/s, *p* = 0.002). Like bimanual dexterity, attending surgeons had higher dominant to non-dominant ratio usage when compared to residents and is statistically significant during gastric pouch creation (1.39 vs. 0.85, *p* = 0.036).

**Table 4:** Subgroup analysis of resident—attending cases categorized based on task group

	Non-dominant instrument speed (cm/s)			Dominant instrument speed (cm/s)			Bimanual dexterity			Workspace volume rate (cm^3^/s)		
	Resident	Attending	*p*	Resident	Attending	*p*	Resident	Attending	*p*	Resident	Attending	*p*
Dissection	2.33 (1.07, 3.59)	3.66 (3.11, 4.21)	**0.023**	1.91 (0.61, 3.21)	3.99 (3.4, 4.58)	**0.002**	0.87 (0.52, 1022)	1.37 (1.03, 1.7)	0.111	11.23 (0.79, 21.66)	28.2 (21.64, 34.74)	**0.007**
Creation of gastric pouch	3.75 (1.92, 5.59)	3.91 (3.45, 4.36)	0.786	3.8 (1.51, 6.1)	4.78 (4.28, 5.29)	0.154	0.85 (0.39, 1.31)	1.39 (1.15, 1.62)	**0.036**	26.78 (18.16, 35.4)	42.95 (33.1, 52.79)	0.059
Measurement	4.49 (2.5, 6.48)	7.77 (7.11, 8.43)	** < 0.001**	5.15 (2.06, 8.23)	7.40 (6.23, 8.56)	0.095	0.86 (0.42, 1.29)	0.89 (0.72, 1.06)	0.853	75.4 (12.56, 138.25)	76.02 (61.43, 90.61)	0.725
Division of mesentery	3.27 (1.75, 4.78)	4.36 (3.8, 4.92)	0.09	2.49 (1.46, 3.52)	4.41 (3.65, 5.16)	**0.02**	0.94 [0.06, 1.79]	1.11 [0.8, 1.43]	0.117	33.59 [22.59, 64.12]	48.09 [28.3, 83.62]	0.314
Staple anastomosis	3.49 (1.94, 5)	4.00 (3.41, 4.59)	0.428	3.11 (0.56, 5.65)	3.95 (3.22, 4.69)	0.338	0.54 (0.12, 0.96)	1.62 (0.26, 2.98)	0.398	24.14 (16.23, 32.05)	29.43 (22.06, 36.79)	0.896
Enterotomy closure	4.24 (3.11, 5.38)	4.74 (4.06, 5.42)	0.43	3.39 (2.75, 5.16)	4.30 (3.71, 4.89)	0.091	0.9 (0.7, 1.09)	1.35 (0.93, 1.78)	0.185	15.18 [7.84, 16.76]	12.23 [7.94, 17.22]	0.818
Defect closure	5.05 (4.44, 5.66)	5.33 (4.78, 5.89)	0.552	4.13 (3.18, 5.09)	4.85 (4.38, 6.31)	0.127	0.93 (0.78, 1.09)	1.38 (1.1, 1.66)	0.059	15.6 (9.86, 21.35)	25.27 (19.47, 31.09)	0.07
Hand-sewn anastomosis	4.23 (3.22, 5.24)	4.45 (4.05, 4.85)	0.618	3.96 (2.75, 5.16)	4.48 (4.10, 4.87)	0.241	1.1 (0.84, 0.135)	1.21 (1.11, 1.3)	0.305	9.06 (4.76, 13.37)	7.16 (5.7, 8.63)	0.321

Bold value indicates statistical significance

Results reported as means (95% CI) and *p* values calculated using independent *t* test unless indicated by * which are nonparametric data represented as median [IQR] and *p* values are calculated via Mann–Whitney *U* test

Trends of improvement in OPIs were also observed with increasing experience of trainees suggestive of a dose–response curve. The percentage of total active console time was higher in fellows compared to residents during Measurement (82.67% vs. 52.61%, *p* = 0.037) and Stapled Anastomosis (72.77% vs. 56.46%, *p* = 0.038). Similarly, fellow’s non-dominant instrument pathlength speed was noted to be improved significantly during measurement (8.11 cm/s vs. 4.49 cm/s, *p* = 0.014) and defect closure (6.14 cm/s vs. 5.05 cm/s, *p* = 0.038). No significant changes were noted for bimanual dexterity or workspace volume. There was no association between trainee participation (i.e., active console time) and case complexity (i.e., primary vs. revision cases) or patient characteristics (such as BMI).

## Discussion

The application of OPIs for the assessment of surgical skills during robotic bariatric surgery has received little attention. In this study, we have demonstrated that significant differences exist in OPIs between experienced and trainee surgeons during key steps of r-RYGB. Our findings reveal that experienced surgeons generally exhibit higher bimanual dexterity and faster non-dominant hand speeds, suggesting greater proficiency and efficiency in this procedure. However, the nuances of our results indicate that different OPIs may vary depending on the step performed during an operative step. For example, in the step of gastric pouch creation, attending surgeons showed similar bimanual dexterity but slower non-dominant hand speed during precise instrument positioning, suggesting that this step may require a more deliberate control of the non-dominant hand approach when compared to dissection in other parts of the procedure. A general trend of lower bimanual dexterity was observed in trainees across most surgical steps, suggesting a potential area for focused skill development. Additionally, specific steps such as measurement and mesenteric defect closure showed higher involvement from trainees, as evidenced by their total active console time. Understanding these differences in OPIs can help better discern the experience levels of surgeons and identify areas to target for training, ultimately enhancing surgical outcomes.

Our study’s findings highlight the varying levels of trainee involvement in different steps of r-RYGB suggesting a potential opportunity for targeted training and skill development in areas, where trainees can safely take on more responsibility. Previous literature supports the use of active console time as an objective measure of trainee participation in robotic surgery (autonomy) and has found that the percentage of active control time decreases for complex cases and for less experienced trainees [20]. Clanahan et al. (2023) further demonstrated the effectiveness of targeted educational resources in promoting trainee participation in robotic bariatric procedures, leading to increased active control time and more active operating at the console [21]. Chen et al. (2018) also emphasized the value of OPIs in measuring surgeon performance during robotic vesicourethral anastomosis and used OPI to guide curriculum development for trainees [22]. Our results further validate OPIs as a useful tool for assessing surgical autonomy and skill progression. By identifying steps with greater trainee involvement, educators can tailor training programs to enhance learning opportunities and gradually increase trainees’ autonomy in more complex tasks, ultimately improving their proficiency in robotic-assisted procedures.

We observed that experienced surgeons exhibited higher bimanual dexterity when compared to trainee surgeons during certain key steps of r-RYGB. This finding aligns with the literature. Hung et al. (2018) found that experts exhibited a greater ratio of dominant to non-dominant instrument path length distance when compared to novices during the dissection steps, but not during suturing steps [18]. This further supports the notion that experienced surgeons possess better efficiency of non-dominant hand during dissection steps, however, during suturing steps, it requires more balanced use. Moreover, Hung et al. (2019) observed that super-experts (median cases of 3000) used their non-dominant hand more efficiently during critical surgical steps of RARP, resulting in higher bimanual dexterity [13]. Similar findings were supported during robotic-assisted thoracic surgery demonstrating a strong correlation between bimanual dexterity and efficiency in robotic-assisted thoracic surgery [14]. Our study contributes to this body of knowledge by illustrating that even within the specific context of r-RYGB, bimanual dexterity remains a critical determinant of surgical skill, which is a skill domain validated in OSATS and GEARS. We observed that experienced surgeons not only demonstrated higher bimanual dexterity ratios, but also adjusted hand usage in a step-specific manner, reflecting a sophisticated understanding and application of bimanual coordination to optimize surgical outcomes.

Our findings of path length speed of the instruments showed that attending surgeons were generally more efficient with their instrument use with faster speed than trainees; similar improvements were also noted with increasing experience of trainees. However, some observed differences were step-dependent and varied for the dominant and non-dominant hand. Similar findings were reported by Ghodoussipour et al. (2021) in their study using OPIs as an objective assessment during robotic partial nephrectomy. They have found that experts had less usage of the third arm instrument and slower movement during specific steps such as opening Gerota’s fascia, with overall slower path length speed for the non-dominant instrument in experts [23]. In contrast, Nguyen et al. (2019) demonstrated that the path length speed of both dominant and non-dominant hands was faster for the experts (> 100 cases) compared to intermediates (< 100 cases) and novices (no experience), while performing simulated tasks [24]. It remains unclear whether these differences could be attributed to technical efficiency or other confounding factors such as case complexity and patient factors.

During our cohort of r-RYGB cases, attending surgeons demonstrated a more confined workspace using both hand controllers during suturing steps, suggesting greater efficiency in the use of space, and precision in their instrument maneuvers. Although very little literature exists on the study of workspace volume regarding surgical experience, Jarc et al. (2017) explored workspace volume as an objective metric during robotic surgery. They utilized master workspace range (MWR) as one of the conventional metrics, which is defined as 85% of the larger of two radii representing the distance between the average hand position and each sampled position. This metric provides the range of motion during surgery in three dimensions [25]. In relation to our study’s workspace volume, the concept MWR could provide insights into how experienced surgeons may utilize a more confined workspace efficiently, potentially leading to a smaller workspace volume. This aligns with the idea that expert surgeons might use less space during surgery, reflecting their proficiency and precision in maneuvering surgical instruments. However, this variable is still relatively new and requires further validation and research to fully understand its implications and to explore how the insights derived from these metrics can inform actionable improvements in surgical training and performance. While these metrics provide valuable measures of efficiency, currently it should not be directly interpreted as prescriptions for action, such as increasing speed or reducing workspace, without further context-specific validation.

One of the limitations of this project is the relatively limited sample size we were able to capture during the study period. This limitation may impact the generalizability of the findings and may not fully capture the variability in performance among a larger, more diverse group of trainees and surgeons. Additionally, the study focuses solely on console-based hand controller metrics, which, while providing valuable insights, do not encompass the full spectrum of skills and competencies required for robotic surgery. Factors such as decision-making and intraoperative judgment, which are crucial for surgical proficiency, are not captured by these metrics. Additionally, the statistical analysis assumes that the surgical steps are independent variables, when in fact several instances of the subtasks can occur within these steps. Therefore, there may be an unknown relationship regarding order and revisiting of these subtasks. Furthermore, the study does not directly consider the observed OPIs in the context of case complexity and patient factors. Incorporating OPIs with these factors would provide a more comprehensive understanding of the clinical relevance and impact of these metrics on surgical quality and effectiveness.

In future studies, we aim to expand our research by including a larger and more diverse cohort of experienced and trainee surgeons. This will allow us to validate our findings on a broader scale and further explore the nuances of kinematic recording in robotic surgery. Additionally, we plan to investigate the correlation between surgeon-specific OPIs and patient outcomes, building on promising work in urology that has demonstrated the potential of skill metrics to predict urinary continence recovery following robot-assisted radical prostatectomy using machine learning applications [4, 26]. The integration of artificial intelligence and machine learning techniques [27, 28] could further enhance our ability to analyze complex surgical data and uncover meaningful patterns related to surgical performance and patient outcomes. Ultimately, our goal is to leverage OPIs to develop objective assessment tools that not only facilitate robotic learning for trainees but also aid in refining surgeon proficiency overall.

## Conclusion

In conclusion, OPIs serve as a crucial tool in distinguishing the technical proficiency of experienced surgeons from trainees during r-RYGB surgery. The variability of OPIs across step-specific steps underlines the importance of tailoring these indicators to the complexity of each surgical phase, such as dissection, creation of the gastric pouch, and hand-sewn anastomosis. Furthermore, the wide variability in OPIs necessitates a careful selection of metrics that accurately assess surgical proficiency. By comparing the OPIs of trainees with those of experienced surgeons, these indicators not only gauge trainees’ readiness but also provide valuable feedback for their progression in mastering robotic surgery. Hence, OPIs hold significant potential in enhancing the training and assessment of surgeons, ultimately contributing to improved surgical outcomes and patient safety.

## Supplementary Information

Below is the link to the electronic supplementary material.

Supplementary file1 (DOCX 17 kb) **Supplemental Table 1** Workspace volume OPI categorized by task and normalized with respect to time and path length, similar statistical significance was noted with the two normalizations.
