# Supplementary material for: Objective performance indicators during robotic Roux-en-Y gastric bypass distinguish experienced from trainee surgeons: a cross-sectional study
Source: Surg Endosc. 2025 Jul 14;39(8):5178–88. doi: 10.1007/s00464-025-11873-4 (PMC12287124; doi:10.1007/s00464-025-11873-4)
Supplement: Supplementary file 1 — Supplementary file1 (DOCX 17 kb) Supplemental Table 1 Workspace volume OPI categorized by task and normalized with respect to time and path length, similar statistical significance was noted with the two normalizations. [file 464_2025_11873_MOESM1_ESM.docx]

|  | Workspace Volume (cm^3) | | | Normalized Workspace Volume per time (cm3/s) | | | Normalized Workspace Volume per pathlength (cm3/cm) | | |
| --- | --- | --- | --- | --- | --- | --- | --- | --- | --- |
|  | Trainee | Attending | p | Trainee | Attending | p | Trainee | Attending | p |
| Dissection | 2451.5 [667.9, 6617.2] | 6527 [2459, 15780.3] | **0.035** | 14.8 [9.57, 42.06] | 27.96 [13.86, 41.05] | 0.18 | 2.9 [1.8, 9.7] | 4.5 [2.4, 5.8] | 0.33 |
| Creation of Gastric Pouch | 10856.9 [8408.3, 16456.9] | 19809.8 [11635.2, 23761.8] | 0.068 | 34.1 [21.7,54.0] | 35.7 [27.4, 54.0] | 0.47 | 5.5 [3.3, 8.8] | 6.0 [4.0, 9.3] | 0.62 |
| Measurement | 9721.7 [3569.55, 21929.6] | 9912.2 [5991.6, 14261.2] | 0.919 | 76.5 [48.6, 110] | 77.3 [39.8, 106.4] | 0.73 | 6.4 [4.8, 8.7] | 5.0 [3.6, 7.4] | 0.255 |
| Division of mesentery | 2056.4 [1077.8, 6306.9] | 2545.5 [2078.1, 4784] | 0.378 | 38.3 [27.1, 64.8] | 48.1 [28.3, 83.6] | 0.41 | 7.6 [6.5, 11.6] | 7.5 [5.1, 14.5] | 0.748 |
| Staple Anastomosis | 4189.3 [3382.5, 10647.4] | 6077.4 [3601.9, 10544.7] | 0.888 | 28.7 [18.2, 45.5] | 24.6 [17.4, 34.0] | 0.34 | 5.0 [4.0, 7.3] | 4.6 [2.9, 5.3] | 0.146 |
| Enterotomy Closure | 6950.2 [4485.6, 12792.6] | 4698 [3252.2, 8650.3] | 0.149 | 16.7 [10.8,22.2] | 12.2 [8.0, 17.2] | 0.18 | 2.1 [1.7, 2.6] | 1.6 [1.3, 3.0] | 0.327 |
| Defect Closure | 12570.4 [6718.7, 20659.4] | 12193.1 [7303.1, 1776.6] | 0.611 | 22.9 [16.0, 37.3] | 20.7 [14.9, 33.2] | 0.72 | 2.6 [1.9, 3.8] | 2.6 [2.2, 3.7] | 0.718 |
| Hand Sewn Anastomosis | 9298.8 [6496.8, 10962.7] | 9050.5 [6189.5, 9711.2] | 0.461 | 10.9 [5.4, 15.6] | 5.96 [5.1, 9.3] | **0.027** | 1.3 [0.8, 2.1] | 0.8 [0.6, 1.2] | **0.019** |
| Values are reprsented as Median [IQR] and p-value are calculated via Mann-Whittney U test | | | | | | | | | |

Supplemental Table 1. Workspace Volume OPI categorized by task and normalized with respect to time and pathlength, similar statistical significance noted with the two normalization.
